# Unusual congenital polydactyly in mini-pigs from the breeding group of the Institute of Cytology and Genetics (Novosibirsk, Russia)

**DOI:** 10.18699/VJ21.074

**Published:** 2021-10

**Authors:** S.V. Nikitin, S.P. Knyazev, V.A. Trifonov, А.А. Proskuryakova, Yu.D. Shmidt, K.S. Shatokhin, V.I. Zaporozhets, D.S. Bashur, E.V. Korshunova, V.I. Ermolaev

**Affiliations:** Institute of Cytology and Genetics of the Siberian Branch of the Russian Academy of Sciences, Novosibirsk, Russia; Novosibirsk State Agrarian University, Novosibirsk, Russia; Institute of Molecular and Cellular Biology of the Siberian Branch of the Russian Academy of Sciences, Novosibirsk, Russia; Institute of Molecular and Cellular Biology of the Siberian Branch of the Russian Academy of Sciences, Novosibirsk, Russia; Novosibirsk State Agrarian University, Novosibirsk, Russia; Novosibirsk State Agrarian University, Novosibirsk, Russia; Institute of Cytology and Genetics of the Siberian Branch of the Russian Academy of Sciences, Novosibirsk, Russia; Institute of Cytology and Genetics of the Siberian Branch of the Russian Academy of Sciences, Novosibirsk, Russia; Institute of Cytology and Genetics of the Siberian Branch of the Russian Academy of Sciences, Novosibirsk, Russia; Institute of Cytology and Genetics of the Siberian Branch of the Russian Academy of Sciences, Novosibirsk, Russia Novosibirsk State Agrarian University, Novosibirsk, Russia

**Keywords:** polydactyly, multi-fingeredness, lateral and medial position, mini-pigs of ICG SB RAS, recessive inheritance, incomplete penentrance, полидактилия, многопалость, латеральное и медиальное положение, мини-свиньи ИЦиГ СО РАН, рецессивное наследование, неполная пенетрантность

## Abstract

The article describes a new phenomenon in the breeding group of mini-pigs at the Institute of Cytology and Genetics (ICG, Novosibirsk): polydactyly (extra digits), which is unusual because the additional digits are situated at the lateral surface of legs or at the lateral and medial ones. This anomaly was f irst found here in 2017 in adult animals intended for culling due to incorrect positioning of the legs caused by f lexor tendon laxity and resulting in weight-bearing on the palmar surface of the proximal phalanges (“bear’s paw”). Therefore, the polydactyly of mini-pigs has a pronounced negative selection effect. A visual survey of the livestock was conducted, and a description of the detected anomaly was compiled. The polydactyly in mini-pigs is a stand-alone trait and is not part of any syndromes. Individuals with polydactyly may have extra digits either on pectoral or on pectoral and pelvic limbs. On thoracic limbs, there may be either one lateral digit or a lateral digit and a medially located rudimentary hoof let. On pelvic limbs, only lateral extra digits can occur. Anatomical and morphological analyses showed that the lateral extra digit is an anatomically complete (“mature”) structure, whereas the medial rudimentary digit consists of only a hoof let without other structures characteristic of normal digits. Cytological examination revealed no specif ic karyotypic features, except for Robertsonian translocation Rb 16;17 previously reported for the mini-pigs of the same livestock. Cytological f indings indicated that the polydactyly and Robertsonian translocation are not linked genetically. Genealogical analysis and results of crosses are consistent with a working hypothesis of recessive inheritance of the trait. Overall, the study shows that this type of polydactyly is anatomically and morphologically unique and not typical of Sus scrofa. In this species, only polydactyly types with medial accessory toes have been described and are usually inherited as a dominant trait with incomplete penetrance. In our case, the results of test crosses indicate recessive inheritance of the trait with varying expression and incomplete penetrance, because of which poorly expressed phenotypes are not visually detectable.

## Abstract (ru)

Приведено описание нового для мини-свиней селекционной группы Института цитологии и генетики (ИЦиГ) СО РАН феномена – полидактилии (многопалости). Она отличается от описанных ранее у Sus scrofa проявлений этой аномалии тем, что дополнительные пальцы располагаются либо на латераль- ной стороне конечностей, либо и на латеральной, и на медиальной. Аномалия впервые была обнаружена в 2017 г. у взрослых животных, предназначенных для выбраковки по причине неправильной постановки ног («медвежья лапа»). Таким образом, полидактилия мини-свиней ИЦиГ имеет явно выраженный негативный селекционный эффект. Проведено визуальное обследование поголовья и составлено описание этой анома- лии. Полидактилия мини-свиней ИЦиГ является изолированной и не входит в состав каких-либо синдромов. Особи с полидактилией могут иметь дополнительные пальцы или на грудных, или на грудных и тазовых ко- нечностях. На грудных конечностях могут присутствовать либо по одному латеральному дополнительному пальцу, либо латеральный палец и медиально расположенное зачаточное копытце. На тазовых конечностях встречаются только латеральные дополнительные пальцы. Анатомо-морфологические исследования по- казали, что латеральный дополнительный палец – анатомически достаточно полноценная структура, тогда как медиальный зачаток представлен только копытцем без остальных, свойственных нормальным пальцам, структур. Цитологическое исследование не выявило кариотипических особенностей, за исключением ранее описанной для мини-свиней ИЦиГ робертсоновской транслокации Rb 16;17. Впрочем, результаты исследо- ваний не указывают на сцепление полидактилии и робертсоновской транслокации. Генеалогический анализ и результаты скрещиваний позволяют принять в качестве рабочей гипотезы предположение о рецессивном наследовании признака. В целом исследование показало, что данная форма полидактилии анатомически и морфологически уникальна и не типична для вида S. scrofa, у которого ранее были описаны только формы полидактилии с медиальными дополнительными пальцами, как правило, наследуемые по доминантному типу с неполной пенетрантностью. В нашем случае результаты анализирующих скрещиваний указывают на рецессивное и, возможно, немоногенное наследование признака с варьирующей экспрессией и неполной пенетрантностью, из-за которой слабо выраженные мутантные фенотипы визуально не фиксируются.

## Introduction

The breeding group of mini-pigs at the Institute of Cytology and Genetics of the Siberian Branch of the Russian Academy of Sciences (ICG SB RAS), Novosibirsk, is rather small. Accordingly, there is continuous inbreeding, which results in the appearance of homozygotes for recessive mutations (Nikitin et al., 2014). In late 2017, a new phenomenon was registered in mini-pigs at the ICG SB RAS: polydactyly (extra digits). This anomaly has long attracted the attention of researchers, and in the XVII century its hereditary nature was already identified (Lange, Muller, 2017). Polydactyly can have both atavistic and teratological nature. In the former case, it means complete or partial restoration of a digit(s) lost by the taxon in the course of evolution; in the second, it results from disruptions of normal ontogenesis (Wiesner, Wheeler, 1979). Extra digits may be located on one or more limbs, and their separation from the rest of the digits may be complete or incomplete (Lange, Muller, 2017). It was reported that there is no specific gene that determines the development of a standard or excessive set of digits, but this trait is determined by pleiotropic and polygenic mechanisms as well as various mutations in gene networks that regulate the formation of limbs (Lange, Muller, 2017). Polydactyly may be either a stand-alone abnormal developmental feature (isolated polydactyly) or a sign of a syndrome (syndromic polydactyly) (Gorbach et al., 2010).

The following isolated types of polydactyly are distinguished: 1. Preaxial. The extra digits are located in front of the medial axis of the limb, that is, in front of the first digit (medial position of the extra digit). 2. Postaxial. The other digits are located behind the medial axis, behind the fifth digit (little finger) (lateral position of the extra digit). 3. Central. The rarest, not pre- and not postaxial type. In Sus scrofa pigs, polydactyly was first described more than a hundred years ago (Gorbach et al., 2010). Several types of preaxial polydactyly with incomplete dominance are currently known in this species, including the wild boar (Ptak, 1963; Malynicz, 1982; Gorbach et al., 2010). Preaxial polydactyly with a possible recessive type of inheritance was described relatively recently (Gorbach et al., 2010). It was suggested that this form may be controlled by genes LMBR1, EN2, HOXA10–13, GLI3, WNT2, WNT16, and/or SHH, located on porcine chromosome 18 (Gorbach et al., 2010). It is interesting to note that multi-toed feral pigs are common in Cuba and the neighboring islands, where the second digit is divided into two or three (five- or six-toed animals). This anomaly is accompanied by the so-called “bear’s paw” when the animal stands not on two (the norm) but rather on four digits (Ivanchuk, 2011). In addition, polydactyly was also found in Kuban flood-meadow pigs (Kudryavtsev, 1948).

The purpose of this publication is to describe the polydactyly found in 2017 in the breeding group of mini-pigs at the ICG SB RAS

## Materials and methods

The study includes data on 82 individuals from the breeding group of mini-pigs at the ICG SB RAS. Among them: 1) 9 adult animals and 14 newborn piglets, to describe external manifestations of the polydactyly; 2) 2 individuals – a mature sow and a 5-day-old piglet – for anatomical and morphological analyses; 3) 36 adults and 44 newborn piglets from seven litters with the manifestation of polydactyly, to build a genealogical tree. During visual examination of the piglets with polydactyly, its presence was determined by the number of hooves on the fore and hind limbs of an individual. The anatomical examination was carried out according to generally accepted methods (Glagolev, Ippolitova, 1977; Lebedev, Zelenevsky, 1995). Anatomical examination was carried out according to generally accepted methods. When constructing the genealogical scheme, we assumed a single source of polydactyly: a common ancestor, i. e., for each pair of polydactyly carriers, by tracing the pedigrees in the direction of earlier generations, we found the most recent common ancestor. Statistical analysis of the results of crosses was carried out by a generally accepted method (Lakin, 1990).

For cytogenetic analysis, four individuals underwent biopsies of the auricle tissue (less than 10 mm in size). From the biopsy material, fibroblast cultures were obtained according to methods of A.S. Graphodatsky et al. (1988) with modifications (Beklemisheva et al., 2016). Suspensions of metaphase cells were prepared from actively dividing cultured fibroblasts by a previously published method (Stanyon, Galleni, 1991). GTG-differential staining was performed according to the standard method (Seabright, 1971).

## Results

**Visual analysis**

**Fig. 1.**
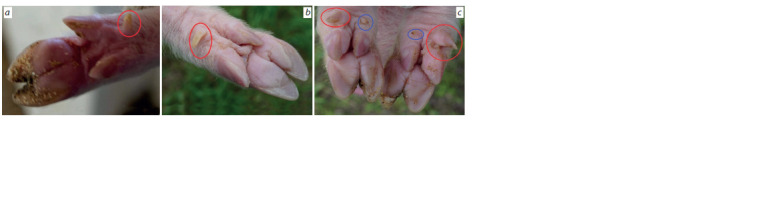
Illustration of polydactyly phenotypes in piglets among the mini-pigs at the ICG SB RAS. Lateral extra digits are indicated by red circles, and medial digits are highlighted by blue circles. a – the f irst phenotype; b – the second phenotype; c – the third phenotype (forelimbs).

Usually, pigs of the S. scrofa species have four toes: the 2nd, 3rd, 4th, and 5th and, respectively, four hooves, two of which (the 3rd and 4th) are supporting (Sokolov, 1979). In polydactyly, there should be more than four toes (hooves) on a pig’s leg. In mini-pigs at the ICG SB RAS, polydactyly has the following phenotypic characteristics (Fig. 1):

1. No other anomalies accompany it. That is, the polydactyly is isolated. 2. The number of extra hooves on an individual limb is either 1 or 2. Accordingly, the total number of hooves on one limb is either 5 or 6. 3. Extra hooves are present either on the two pectoral limbs or on all four. 4. The extra hooves are symmetrical on a pair of limbs. The number of extra hooves on both thoracic limbs is either 1 and 1 or 2 and 2; on the pelvic limbs, either 1 and 1, or none. 5. With two extra hooves on the forelimbs, a larger claw-like hoof is located laterally, and a much smaller hoof is situated medially. 6. With two extra hooves on both thoracic limbs, there is one lateral extra hoof on the pelvic limbs. With one extra hoof on the thoracic limbs, there are no extra hooves on the pelvic limbs. 7. It is accompanied by a “bear’s paw” when an animal with an extra digit stands on all four digits (2nd, 3rd, 4th, and 5th), not on two central digits (3rd and 4th) as is typical of pigs. With extra digits on the pelvic limbs, their incorrect positioning leads to an overgrowth of the hoof horn and lameness. The polydactyly in the mini-pigs at the ICG SB RAS is characterized by a variation of the size of the lateral extra digits among same-age individuals; visually, the length differs 2–3-fold. In general, the variation of the trait is represented by three distinct phenotypes (see Fig. 1): 1. Lateral extra hooves on the thoracic limbs, whose length in newborns is ~1 mm. 2. Lateral claw-like extra hooves on the thoracic limbs, the length of which in newborns can reach 3 mm. 3. Lateral extra hooves of ≥ 3 mm size on the thoracic limbs and medial extra hooves in newborns in the form of a horny tubercle ~1 mm high; at the same time, there are lateral extra hooves on the pelvic limbs.

Polydactyly of mini-pigs at the ICG SB RAS is not typical for the species Sus scrofa, which has preaxial relics (Malinich, 1982; Gorbach et al., 2010). Animals with the first polydactyly phenotypes have lateral accessory hooves, which can be considered as isolated postaxial polydactyly. For the third phenotype is characterized by the simultaneous presence of lateral and embryonic medial accessory hooves, i. e. polydactyly with two additional toes is both pre- and postaxial.

**Anatomical and morphological analysis**

**Fig. 2.**
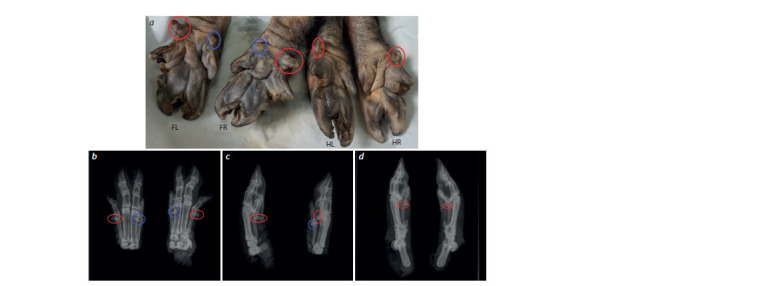
Limbs of an adult sow of the polydactyly phenotype 3. Lateral digits are highlighted by red circles; medial digits are highlighted by blue circles (in panels a–c). Panel a shows a photograph of the limbs: FL – front left; FR – front right; HL – rear left; HR – rear right. Panels b–d present radiographs of the limbs: anterior (left-left and right-right, respectively) in the dorsoventral projection (b), anterior in the lateral view (c), and posterior at the lateral point (d), respectively

When we examined the anatomical material collected from a mature sow with polydactyly of the third phenotypic type, extra digits were visible in the distal part of each of the four limbs. The extra digits were well developed and had a pronounced stratum corneum and an anatomical configuration corresponding to the normally developed digits in pigs but with signs of atrophy. On the 2nd digit of the left hind leg, an overgrown deformed hoof was visible. The rest of the hooves with extra digits had a pathological shape due to an anomaly of the limbs (Fig. 2, a).

A radiograph of distal thoracic extremities revealed their tendency to perform the function of additional support (see Fig. 2, b–d ). Note the relative topographical position of the phalanges of the extra digits on both thoracic limbs. They were found to be located at the distal ends of fifth metacarpal bones, proximal to the first interphalangeal joint of the forelimb’s fifth digit. From the lateral surface, the radiograph shows (in an especially clear image of the left forelimb) the presence of phalanges of the lateral extra digit, with a confirmed anatomical basis for the functions of support, blood supply, innervation, and other trophic functions.

**Fig. 3.**
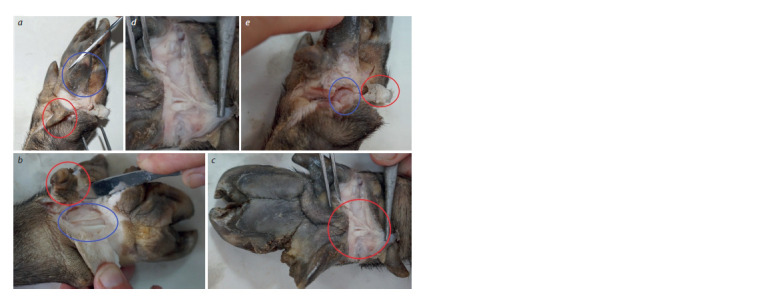
Analytical preparation of an extra digit from a thoracic limb of an adult female. а – the f irst stage of the preparative procedure with the separation of the digit; the wear and tear of the hoof horn is visible on the normal lateral (in a blue circle) and extra (in a red circle) digits; b – the second stage of preparation, the histological base (in a blue circle) of the extra digit (encircled in red) is visible; c – the third stage of preparation. The tendon (in the red circle) of the ligament of the extra digit is visible; d – an enlarged image of the tendon; e – dissection of the joint (in the outgrowth indicated by the blue circle) of the extra digit without the horn cover (in the red circle).

In the examination of a dissected area above the lateral extra digit, a well-formed connective tissue ligament (tendon) was noted on all limbs, which is characteristic of anatomical and topographic structures of porcine distal extremities (Fig. 3). The ligaments of the extra digit consist of smooth fibrous connective tissue with morphological features characteristic of a normal pig limb. Phalanx development with a typically formed metacarpophalangeal joint yielded all the necessary components of a proper joint (synovial fluid, joint ligament, and unity of articular surfaces). The sample demonstrates structural units (common tendon) that determine a possible functional purpose of the extra digit.

**Fig. 4.**
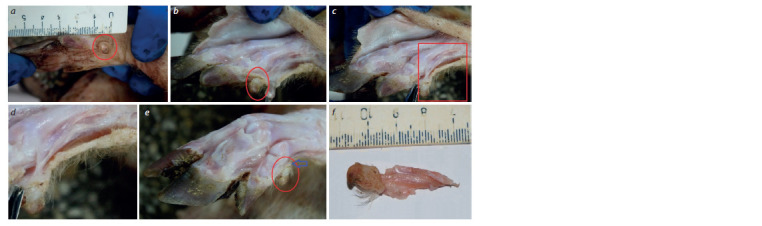
Anatomical and morphological analyses of the thoracic limbs carrying a lateral extra digit in a 5-day-old piglet from the mini-pigs at the ICG SB RAS.

External examination of a 5-day-old piglet revealed extra digits in the distal part of thoracic limbs on the lateral surface, which have spiny protrusions with formed horny layers (see Fig. 1, a and 4, a). Lateral extra digits have a pronounced supporting function, as evidenced by the finding that, five days after birth, there was a noticeable deterioration of the stratum corneum surface. Similar deterioration of the hoof horn on the extra digits also occurred on the limbs of an adult sow (see Fig. 3, a). There is a pronounced caudal orientation of the extra digits, opposite to the direction of the four normal ones, which was also found on the pelvic limbs of the adult sow (see Fig. 2, a). An extra digit on the lateral side of the limb in both the former and latter cases ends in a claw-like hoof; in the adult sow, it was found to be damaged and blunted at the end (see Fig. 3, a and c), and in the piglet, the hoof was still sharp (see Fig. 4, f ). That is, the lateral extra digit serves as an additional support for the “bear’s paw”.

а – size comparison of standard and extra digits (in a red circle or box); b – removal of the skin from the preparation of the limb from a mini-pig with polydactyly (the abnormal lateral digit is in the red circle); c – demonstration of the tendons (indicated by the red circle) that attach the extra digit to the bones of the pastern and the phalanx of a normal digit; d – an enlarged photo of the same tendons; e – demonstration of the bone base of the extra digit on the processed limb of the mini-pig; the blue arrow indicates the joint; f – preparation of the extra digit.

The preparations of digits from a 5-day-old piglet, including a lateral extra digit, featured a bone base (see Fig. 4, b). On the lateral surface separated from the skin, phalanges of the extra digit and a coarse-fibrous-connective-tissue ligament, which is the structural and functional unit of the extra digit, can be observed. On the preparation of the distal limb, the interphalangeal joints of all digits are visible, including the extra digit (see Fig. 4, c and d ). The interphalangeal joint of the extra digit is well-pronounced (see Fig. 4, e). The separated dissected extra digit of the thoracic limb from the 5-day-old piglet had all the characteristic morphological features (see Fig. 4, f ).

Overall, the anatomical analysis of the lateral extra digits from mini-pigs of the ICG SB RAS revealed that in this selection group, a potentially functional structure, i. e., a lateral extra digit with a pronounced bone support base, is present. These data suggest that with a possible load of the limb on it, there is formation of qualitative reference indicators in ontogenesis. On the contrary, the medial extra digit does not have such a supporting bone base and is represented only by the hooflet. Its position in the first digit (thumb) lost in the process of evolution points to incomplete materialization of the second extra digit wherein the extra digit “replaces” the lost thumb.

**Genealogical and genetic analyses**

**Fig. 5.**
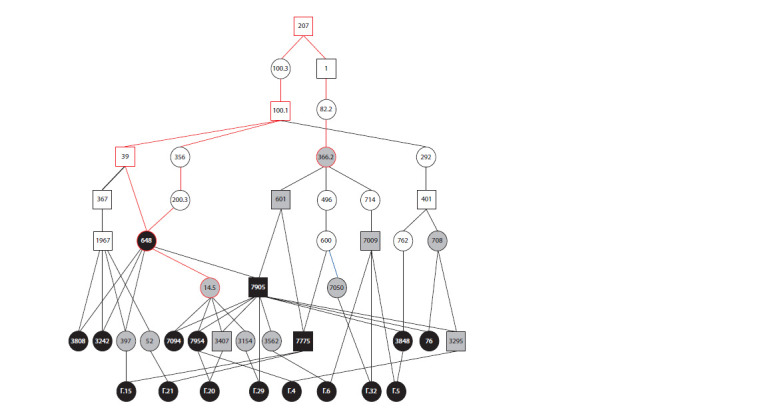
Scheme of polydactyly transmission pathways in the breeding group of mini-pigs at the ICG SB RAS. Squares indicate males; circles indicate females; black symbols indicate individuals with polydactyly; grey symbols indicate normal phenotype (the carriage of the trait’s genetic factor was conf irmed by crosses); white symbols indicate normal phenotype individuals (with unconf irmed carriage of the trait). Red color indicates the ancestors on which the pedigrees of individuals with the manifestation of polydactyly converged and the path between these ancestors.

A genealogical scheme based on the principle of the lowest common ancestor of a pair of individuals was constructed to clarify the source of the polydactyly in the breeding group of mini-pigs at the ICG SB RAS. The scheme shows that all probable ascending lines of polydactyly inheritance converge on a common ancestor: boar No. 207 (Fig. 5). Currently available data are insufficient for objective reliable testing of the hypotheses about the inheritance of the trait. The reason is that only a year after the discovery in the breeding stock of individuals with polydactyly, all newborn piglets were examined to register the presence of this anomaly.

**Fig. 6.**
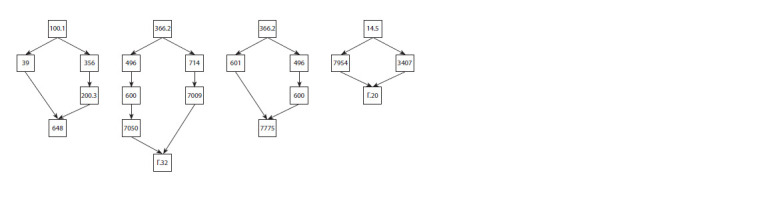
Fragments of pedigrees of individuals manifesting polydactyly. IDs of the animals are indicated in squares (gender is not shown). At the top of the pedigrees, the nearest common ancestors are presented, and at the bottom, probands with polydactyly.

From 2018 to 2020, eight litters with polydactyly were obtained: a total of 51 newborns, 14 of them with extra digits. In two litters, where both parents had a normal phenotype (see Fig. 5), six piglets were born, and in each one polydactyly was present. In six other litters, one of the parents had polydactyly,the other one was normal (but had a parent with polydactyly) (see Fig. 5). They gave birth to 39 piglets, of which 12 had extra digits. The genealogical scheme indicates that in all 16 cases of polydactyly, the pedigrees are connected by an ancestor common to the parents of such an individual (see Fig. 5). Based on the pedigrees (Fig. 6), without resorting to statistical analysis, it is already possible to assume recessive inheritance of the polydactyly in the breeding group of mini-pigs. 

More information about the genetic nature of this anomaly was given by the outcome of test crosses between i) the pigs that were phenotypically normal but heterozygous for the polydactyly factor(s) and ii) the animals that had extra digits. Statistical analysis of the analytical crossbreeding results (see the Table ) revealed that the assumption of monogenic inheritance (in which the offspring would be expected to split according to the phenotype in the ratio of 1:1) is rejected (χ2 = 5.76, d.f. = 1). Probably, what occurs here is recessive polygenic (with one or several major genes) inheritance, although it is impossible to rule out a monogenic state with incomplete penetrance, which may cause visually undetectable weak expression of the trait. On the other hand, because the numbers of offspring of the two classes were relatively small, it is necessary to set up other crosses for a more accurate assessment of a larger sample of pigs.

**Table.**
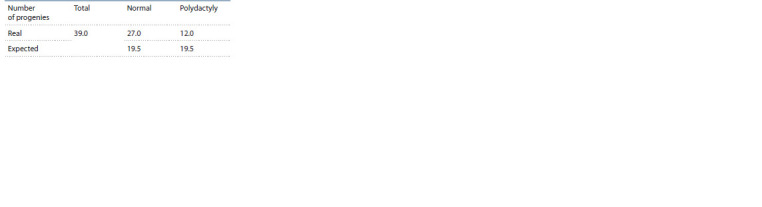
The results of backcrosses between pigs with polydactyly and heterozygote for the genetic factor(s) of polydactyly

**Cytogenetic analysis**

**Fig. 7.**
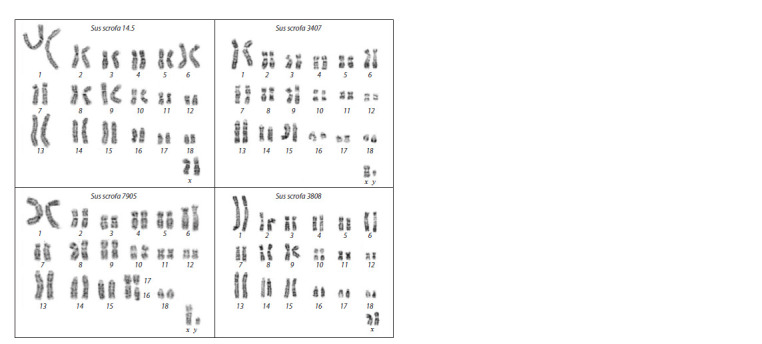
GTG-stained chromosomes of four individuals in the breeding group of mini-pigs at the ICG SB RAS.

To identify specific features of the karyotypes in the breeding group of mini-pigs, this analysis was performed on four individuals. Karyotypes were obtained for the following minipigs: two with the normal phenotype (female No. 14.5 and male No. 3407) and two with polydactyly (female No. 3808 and male No. 7905) (Fig. 7).

Based on the GTG-banding data, it was demonstrated that the karyotypes of three studied individuals (IDs 14.5, 3407, and 3808) do not differ from the previously published conventional karyotypes of Sus scrofa in the number (2n = 38), morphology, and the GTG band pattern of chromosomes (Graphodatsky et al., 2020) (see Fig. 7). In the male with polydactyly (pig No. 7905), a Robertsonian translocation (Rb 16;17, 2n = 37) was detected, the occurrence of which among the mini-pigs of the ICG SB RAS was reported earlier (Tikhonov et al., 2010), for example, in its male progenitor with polydactyly (pig No. 207).

## Discussion

The polydactyly that manifested itself in the breeding group of mini-pigs at the ICG SB RAS is unique for S. scrofa. It combines pre- and postaxial types with obvious predominance of the latter. From the point of view of microevolutionary processes, the polydactyly in the mini-pigs at the ICG SB RAS is evidently a new physical feature, namely, the formation of an almost complete lateral extra digit. With maximum expression of this trait, another extra digit, i. e., a rudimentary medial hooflet, is observed at the site of the thumb. In general, S. scrofa is characterized by the medial location of the extra digits previously found in some individuals (Malynicz, 1982; Gorbach et al., 2010); the extra digits seem to “replace” the thumb, although anatomically, these “replacements” can differ very significantly from the thumb (Malynicz, 1982; Gorbach et al., 2010). In general, it seems that the very genetic mechanism underlying the formation of the thumb is disrupted in the cases described earlier. Still, information about its location is preserved in the genome.

On the contrary, in the mini-pigs from the ICG SB RAS with five digits, the location of the extra digit with the corresponding fully formed anatomical and morphological structures is genetically determined at the site of the sixth digit (a “second” little finger). When mini-pigs have another type of the anomaly in the form of “six digits” and the extra little toe, there is a rudimentary hoof in place of the first digit (thumb). Still, all the other structures inherent in normal digits are absent here. As a consequence, the rudimentary hooflet of this second extra digit on the six-toed pectoral limb of a mini-pig is located in the first digit (thumb) of the pectoral limb. We believe that this phenomenon requires further anatomical, morphological, and molecular-genetic studies.

Our analysis of karyotypes by standard cytogenetic methods did not reveal any specific features in our mini-pigs with extra digits, except for the Robertsonian translocation Rb 16;17 in one of the four tested animals; this feature was previously identified in this population (Tikhonov et al., 2010).

Candidate genes that may determine polydactyly in pigs are located on chromosome 18 (Gorbach et al., 2010). According to the obtained GTG-banding data, no inter- and intrachromosomal rearrangements involving this chromosome are present in these mini-pigs.

## Conclusion

**Fig. 8.**
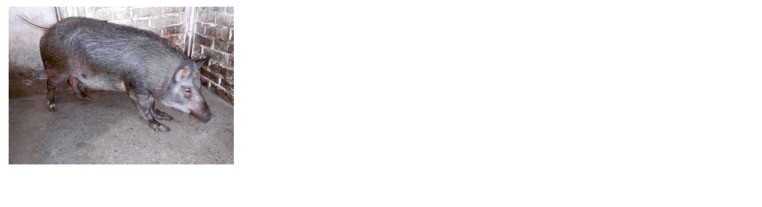
An adult mini-pig female with polydactyly and carpal laxity (“bear’s paw”).

The results of test crosses indicate recessive inheritance of the trait with varying expression and incomplete penetrance, which may also explain why poorly expressed phenotypes are not visually detectable. It is possible that a “bear’s paw” without extra digits, which is not noticeable in newborn piglets, may also represent a sort of polydactyly phenotype. In conclusion, it should be noted that the polydactyly in the mini-pigs at the ICG SB RAS has an apparent selection-negative effect. In animals with a “bear’s paw”, a hoof horn may grow on 3–4 digits, thereby leading to lameness (Fig. 8). Therefore, polydactyly was first detected among the mini-pigs at the ICG SB RAS when we examined animals culled from the breeding stock owing to incorrect leg positioning or lameness

## Conflict of interest

The authors declare no conflict of interest.
